# GNSS Interference Identification Driven by Eye Pattern Features: ICOA–CNN–ResNet–BiLSTM Optimized Deep Learning Architecture

**DOI:** 10.3390/e27090938

**Published:** 2025-09-07

**Authors:** Chuanyu Wu, Yuanfa Ji, Xiyan Sun

**Affiliations:** 1 Information and Communication School, Guilin University of Electronic Technology, Guilin 541004, China; wcy@mails.guet.edu.cn; 2 Guangxi Key Laboratory of Precision Navigation Technology and Application, Guilin University of Electronic Technology, Guilin 541004, China; 3 International Joint Research Laboratory of Spatio-Temporal Information and Intelligent Location Services, Guilin University of Electronic Technology, Guilin 541004, China; 4 GUET-Nanning E-Tech Research Institute Co., Ltd., Nanning 530031, China

**Keywords:** GNSS interference detection, image processing, entropy, deep learning, metaheuristic optimization, signal classification

## Abstract

In this study, the key challenges faced by global navigation satellite systems (GNSSs) in the field of security are addressed, and an eye diagram-based deep learning framework for intelligent classification of interference types is proposed. GNSS signals are first transformed into two-dimensional eye diagrams, enabling a novel visual representation wherein interference types are distinguished through entropy-centric feature analysis. Specifically, the quantification of information entropy within these diagrams serves as a theoretical foundation for extracting salient discriminative features, reflecting the structural complexity and uncertainty of the underlying signal distortions. We designed a hybrid architecture that integrates spatial feature extraction, gradient stability enhancement, and time dynamics modeling capabilities and combines the advantages of a convolutional neural network, residual network, and bidirectional long short-term memory network. To further improve model performance, we propose an improved coati optimization algorithm (ICOA), which combines chaotic mapping, an elite perturbation mechanism, and an adaptive weighting strategy for hyperparameter optimization. Compared with mainstream optimization methods, this algorithm improves the convergence accuracy by more than 30%. Experimental results on jamming datasets (continuous wave interference, chirp interference, pulse interference, frequency-modulated interference, amplitude-modulated interference, and spoofing interference) demonstrate that our method achieved performance in terms of accuracy, precision, recall, F1 score, and specificity, with values of 98.02%, 97.09%, 97.24%, 97.14%, and 99.65%, respectively, which represent improvements of 1.98%, 2.80%, 6.10%, 4.59%, and 0.33% over the next-best model. This study provides an efficient, entropy-aware, intelligent, and practically feasible solution for GNSS interference identification.

## 1. Introduction

Global navigation satellite systems (GNSSs) have become critical infrastructure that supports modern society, with applications that span aviation, maritime navigation, unmanned aerial vehicle autonomous navigation, intelligent transportation, precision agriculture, and many other fields, thus serving as a vital technological foundation for societal operations [1]. However, as GNSS applications continue to expand, the security threats they face are becoming increasingly severe. In high-risk scenarios such as civil aviation takeoff and landing and drone swarm operations, malicious interference can cause navigation signal distortion or even service disruption, which can lead to major safety incidents [2]. GNSS frequency band interference signals can disrupt service functions over vast geographical areas, thereby resulting in positioning errors, speed measurement inaccuracies, and timing anomalies. These issues can range from causing navigation errors to system crashes or even major safety incidents [3]. This interference not only severely degrades system performance but also poses potential threats to low-altitude safety and public security [4]. Therefore, research on the precise identification and classification of GNSS interference signals is highly important for ensuring system reliability, mitigating major risks, and maintaining safety and social stability.

Significantly, within the realm of advanced applications reliant on precise GNSS services, reliable sign detection and interference identification are not merely desirable for system robustness, but constitute critical prerequisites for ensuring autonomous operation and decision-making safety. For instance, in multi-perspective Unmanned Aerial Vehicle object detection tasks, accurate target localization and environmental perception critically depend on trustworthy navigation signals; the presence of GNSS interference can directly degrade the input quality to detection models, jeopardizing both detection accuracy and system safety [5]. In the context of dispatch optimization for Shared Autonomous Vehicles, efficient vehicle positioning and route planning form the foundation for achieving system stability and maximizing overall performance; interference-induced positioning drift or loss can severely compromise the execution of optimal dispatch strategies [6]. Similarly, in LiDAR-based Simultaneous Localization and Mapping systems enhanced by GNSS, the global position constraints provided by GNSS are vital for correcting odometry drift and enhancing map accuracy; interfering signals can significantly undermine the efficacy of GNSS-aided SLAM graph optimization [7]. Therefore, within these critical and safety-sensitive applications, conducting research into the precise identification and classification of GNSS interference signals holds paramount significance for ensuring reliable system operation, mitigating significant risks, and safeguarding public safety and social stability.

In recent years, GNSS jamming technology has trended toward increasing complexity, diversification, and stealthiness in its forms. We can classify the interference into continuous wave interference (CWI), Chirp interference, pulse interference, frequency modulation noise interference (FMI), amplitude modulation noise interference (AMI), and spoofing interference [8]. While traditional CWI effectively suppresses receiver capture and tracking loops because of its concentrated energy and ease of implementation, its steady-state characteristics make it relatively easy to detect. In contrast, chirp jamming leverages rapidly varying spectral characteristics and pulse interference uses instantaneous peak power; both effectively evade conventional filter designs and rapidly cause positioning errors or receiver lock loss. FMI and AMI employ wide-spectrum modulation techniques to mimic environmental noise characteristics, introducing high levels of uncertainty and complexity that increase the information entropy of the signals, thereby significantly increases the probability of misclassification in interference detection. The most challenging type of interference is spoofing interference. Spoofing interference achieves high-fidelity emulation across multiple signal dimensions (time—frequency, structure, power, spatial), making it statistically very similar to authentic signals in terms of measured information entropy or mutual information, and endowing it with extremely strong stealth and deception capabilities [9]. The rapid evolution and diversification of interference techniques resulting in highly complex, uncertain, and high-entropy signal environments, have highlighted the urgent need for efficient detection and precise identification of various interference signals, regardless of whether they are suppression-type or spoofing-type.

The continuous iteration of interference techniques (especially the parameter similarity between deceptive signals and legitimate signals) has led to a double bottleneck for traditional detection methods: First, their classification capabilities are insufficient in complex and entropy-rich electromagnetic environments. Conventional signal quality monitoring (SQM [10]) technology has difficulty distinguishing between different types of interference in interference scenarios, and its role is often limited to detecting the presence of interference signals [11,12]. Second, there are blind spots in the detection of highly realistic deceptive signals, and parameterized deception technology can circumvent detection mechanisms on the basis of traditional metrics such as the carrier-to-noise ratio (C/N_0_) and correlation peak symmetry [13].

Therefore, in the field of GNSS security protection, the intelligent identification and classification of interference signals have become the core research issues for overcoming the current bottlenecks. Robust classification models that are based on deep learning feature extraction can output high-confidence interference-type discrimination results that provide category prior information for adaptive notch filtering, spatiotemporal processing, and multidimensional detection algorithms, thereby enabling the real-time generation of optimal suppression strategies through adaptive parameter configuration. This technical approach achieves rapid and accurate perception and determination of complex interference categories, thus laying a foundation for the construction of a closed-loop defense framework. This approach holds decisive significance for enhancing the availability and continuity of GNSS services in complex electromagnetic environments and ensuring the safe operation of critical infrastructure.

Deep learning, as a powerful data-driven method, has shown great potential in GNSS interference signal classification tasks because of its excellent pattern learning and feature extraction capabilities. It can deeply analyze signal images (such as time—frequency diagrams and eye diagrams) and sequence data characteristics, extract deep features, construct accurate models to effectively identify potential patterns of interference signals and achieve efficient and reliable classification, thus providing new ideas for overcoming the limitations of traditional methods.

Several machine learning-based studies on GNSS interference signal classification (especially spoofing detection) have been conducted. Zhu et al. [14] used support vector machine (SVM) to analyze seven features (SQM, moving variance, moving average, early and late phases, etc.) to detect spoofing signals. Wang [15] and Chen [16] both adopted a method that combines multiple parameters with an SVM to improve spoofing detection performance. Li et al. [17] extracted multiple features, including SQM, C/N_0_, and pseudorange-Doppler consistency, and validated their performance on the open-source deception dataset TEXBAT. For interference pattern recognition, O’Shea et al. [18] explored the application of a convolutional neural network (CNN) in the identification of complex radio signal modulations. Ferre et al. [19] transformed the spectral plots of signals into images, thus converting the interference type classification problem into a spectral plot classification task, and compared the performance of a SVM and a CNN in distinguishing between continuous waves, linear frequency modulation, multitone frequency modulation, pulses, and narrowband frequency modulation interference.

However, existing methods have significant limitations. First, methods that are based on manual features are highly dependent on feature engineering, lack flexibility, and are difficult to generalize. Second, existing machine learning and deep learning models, such as SVM, multilayer perceptron (MLP [20]), CNN [21], long short-term memory (LSTM [22]) and bidirectional long short-term memory (BiLSTM [23]), still have room for improvement in terms of classification accuracy, especially when dealing with multiple types of interference patterns.

To overcome the above limitations, a CNN–BiLSTM–ResNet hybrid model that is based on an improved coati optimization algorithm (ICOA–CNN–BiLSTM–ResNet) for interference signal classification is proposed in this study. The core strategy is to construct an eye diagram dataset of interference signals by using the correlation values of GNSS receivers and convert the interference signal classification problem into an eye diagram image classification problem. This transformation leverages the visual representation to encode the underlying statistical variations and entropy signatures induced by different jamming types.

At the model design level, single-structure deep learning methods face significant limitations: traditional CNN models can extract local spatial features effectively, but they struggle to model long-range global context dependencies; BiLSTM models can capture the temporal dependencies of sequence data, helpful for modeling time-varying information content, but they are still constrained by the vanishing gradient problem when processing extremely long sequences; and deep residual network (ResNet [24]) alleviate the degradation issues of deep networks through skip connections, thereby enhancing feature propagation capabilities, but their primary advantage lies in spatial feature representation, capturing multi-scale informational structures, and they have relatively limited ability to capture complex dynamic patterns in signals that exhibit high temporal entropy. To overcome the inherent shortcomings of the above single models, we designed a CNN–ResNet–BiLSTM hybrid architecture. In this architecture, a CNN acts as a front-end feature extractor to efficiently capture the eye diagram features of interference signals, a ResNet integrates and deepens the features passed by the CNN layer and uses its powerful deep representation ability and the stability of residual learning to learn more robust and abstract discriminative patterns from multilevel features, and a BiLSTM network leverages its bidirectional temporal modeling capabilities to understand the temporal and spatial correlations of signals in detail and capture the unique long-term and short-term dynamic evolution patterns of fraudulent signals. Through innovative model design, this approach effectively combines the spatial feature extraction advantages of CNN, the deep feature abstraction and stability optimization characteristics of ResNet, and the bidirectional temporal modeling capabilities of BiLSTM, thereby significantly improving the classification performance for complex and diverse interference signals.

Despite the outstanding performance of deep learning models, their parameter optimization process still faces significant challenges. Traditional parameter optimization methods such as stochastic gradient descent (SGD) and adaptive moment estimation (Adam) are prone to becoming stuck in local optima and have limited convergence speeds in high-dimensional spaces. Researchers have proposed various novel optimization algorithms to address these issues, but their applications remain limited. For example, the coati optimization algorithm (COA) [25], which was proposed by Dehghani, draws inspiration from the foraging behavior of coatis for search and demonstrates strong local search capabilities. However, it suffers from insufficient global convergence in high-dimensional multimodal problems and is easily constrained by initial population diversity, which makes it unable to find boundary optimal solutions. Braik’s chameleon swarm algorithm (CSA) [26] incorporates the chameleon visual mechanism to enhance exploration, but it overly relies on individual information interaction, which leads to premature convergence and a decline in search activity in the later stages. Liu’s adaptive particle swarm optimization (APSO) [27] improves upon traditional PSO by increasing convergence speed and global performance, but it still struggles to avoid particle diversity decay and faces the risk of premature convergence in nonconvex tasks. The gray wolf optimization algorithm (GWO) [28] is based on the social hierarchy and hunting strategy (α/β/δ guidance) of gray wolves and has strong global search capabilities. However, population homogeneity increases in the later stages, thus limiting the ability to escape from local optima, which is a phenomenon that becomes more pronounced in high-dimensional problems. The whale optimization algorithm (WOA) [29] simulates whale bubble net hunting, with efficient initial exploration but excessive focus on the current optimal solution during the development phase. The imbalance between exploration and exploitation leads to insufficient convergence stability when complex optimization surfaces are faced. Additionally, Yan optimized GRU hyperparameters via the simulated annealing (SA) algorithm [30] as a single-point search strategy, which has low search efficiency and limited optimization capabilities in high-dimensional spaces.

In response to the limitations of the above algorithms, an improved COA (ICOA) is proposed in this study. This algorithm makes the following improvements on the basis of the COA. It uses a logistic–tent chaotic mapping mechanism to generate the initial population, which overcomes the limitations of traditional algorithms in the selection of initial solutions and improves the diversity and uniformity of the population. It also uses an elite perturbation strategy in which controllable perturbations are periodically applied to the current optimal solution to introduce moderate randomness, thus breaking the algorithm’s potential local stability while maintaining the trend toward convergence to the globally optimal region. In addition, we introduce a dynamic weighting mechanism that is based on the original exploration and development stages to enhance exploration capabilities in the early stages and focus on development accuracy in the later stages, thereby balancing global exploration and local development capabilities. By combining the above improvements, the ICOA not only enhances the search efficiency and convergence speed but also demonstrates superior stability and generalization capabilities in avoiding premature convergence and optimizing high-dimensional spaces.

In summary, the main contributions of this study are as follows: (1) A new method for constructing eye diagram datasets on the basis of GNSS receiver correlation values is proposed, which transforms the interference signal classification problem into an eye diagram image classification problem. This transformation efficiently captures the critical information-theoretic characteristics (like signal uncertainty/distribution) of jamming. (2) A hybrid deep learning model (CNN–ResNet–BiLSTM) that integrates the advantages of CNN, BiLSTM, and ResNet to comprehensively model the spatial, temporal, and hierarchical informational entropy of interference signals was designed and verified for interference signal classification. (3) An improved coati optimization algorithm is proposed, which effectively solves the global convergence and local optimality problems in deep learning model hyperparameter optimization. (4) Extensive experiments were conducted on interference signal datasets. The results show that the proposed ICOA–CNN–ResNet–BiLSTM hybrid model achieved a classification accuracy of over 98%, which was significantly better than those of the baseline models (e.g., CNN, LSTM, SVM, and CNN-LSTM [31] models).

The remainder of this paper is organized as follows: Section 2 introduces a mathematical model of interference signals and their eye diagram characteristics, Section 3 details the ICOA–CNN–ResNet–BiLSTM model structure and optimization methods, Section 4 evaluates the model performance on the basis of the interference signal dataset, and Section 5 summarizes the paper.

## 2. Signal Model

**Figure 1:**
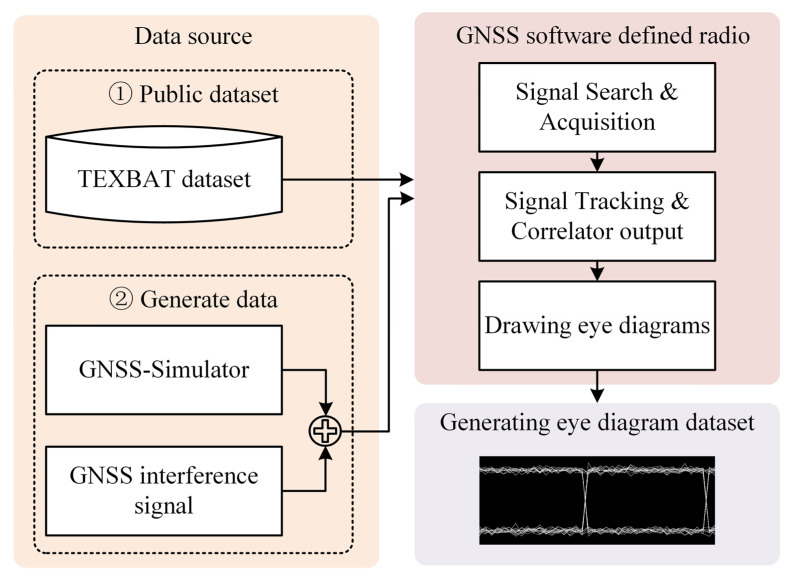
GNSS signal interference eye diagram dataset generation process.

In this chapter, the construction of a signal processing model for GNSS interference classification research is described, which lays a theoretical foundation for subsequent interference feature analysis and deep learning classification algorithms. The model completely describes the entire process from satellite signal transmission, propagation through channels that contain interference and noise, and receiver front-end processing to baseband correlation processing and eye diagram generation (Figure 1). The core goal of this research is to reveal how different interference signals disturb the correlator output values and ultimately manifest as unique anomaly patterns in the eye diagram that correspond to distinct statistical and entropic signatures induced by the jamming.

### 2.1. Signal Reception Model

The baseband signal received by the GNSS receiver antenna is considered. This signal consists of the true satellite navigation signal, jamming signal, and receiver noise and can be expressed by the following mathematical model:

(1) $$r\left(t\right)\;=\;s\left(t\right)\;+\;j\left(t\right)\;+\;n\left(t\right)$$

where $s\left(t\right)\;$ represents the target GNSS satellite signal (GPS L1 as an example). A single visible satellite signal can be expressed as

(2) $$s(t)=\sqrt{P_{s}}\cdot d(t-\tau_{s})\cdot c(t-\tau_{s})\cdot e^{j(2\pi f_{d}t+\phi_{s})}$$

where $P_{s}$ denotes the received signal power, $d\left(t\right)$ denotes the navigation message, $c\left(t\right)$ denotes the pseudorandom code (C/A code), $\tau_{s}$ denotes the signal propagation delay, $f_{d}$ denotes the carrier Doppler shift, and $\phi_{s}$ denotes the carrier initial phase. $j\left(t\right)$ denotes the jamming signal, and $n\left(t\right)$ denotes additive white Gaussian noise (AWGN), whose bilateral power spectral density is *N*_0_/2 and follows a complex Gaussian distribution $n(t)\sim CN(0,N_{0})$.

### 2.2. Receiver Front End and Related Processing Model

The GNSS receiver digitizes and processes the received signal $r\left(t\right)\;$ in the baseband. The key step is correlation processing, in which correlation operations between locally generated replica signals and the received signal are performed to demultiply the signals and obtain observation values such as the pseudorange and carrier phase.

- Local replica signal

(3) $$l_{p}(t)=c(t-\hat{\tau })\cdot e^{j(2\pi \hat{f_{d}}t+\hat{\phi })}$$

where $\hat{\tau }$, $\hat{f_{d}}$, and $\hat{\phi }$ represent the locally estimated code delay, Doppler frequency shift, and carrier phase, respectively.

- Correlator output

The correlation integral is performed within the integration time (1 C/A code cycle):

(4) $$I_{p}(k)+jQ_{p}(k)=\frac{1}{T}\int_{(k-1)T}^{kT}r(t)\cdot l_{p}^{*}(t)\;dt$$

where k denotes the index of the kth integration period and where ^*^ denotes complex conjugation.

- Correlation amplitude (core feature)

(5) $$A_{p}(k)=\sqrt{I_{p}^{2}(k)+Q_{p}^{2}(k)}$$

$A_{p}(k)$ intuitively reflects the strength of the correlation between the local signal and the received signal. Jamming signals significantly alter the statistical characteristics of $I_{p}^{2}(k)$ and $Q_{p}^{2}(k)$, which causes abnormalities in the trajectory and distribution of $A_{p}(k)$.

### 2.3. Interference Signal Model

Each type of interference signal has its own unique mathematical expression, and its effects on eye diagrams are also unique. From changes in frequency and phase to amplitude, different types of interference can cause problems such as spectrum expansion, waveform distortion, and eye diagram closure. This study focuses on the following six types of interference (Table 1): CWI, chirp interference, pulse interference, FMI, AMI, and spoofing interference.

**Figure 2:**
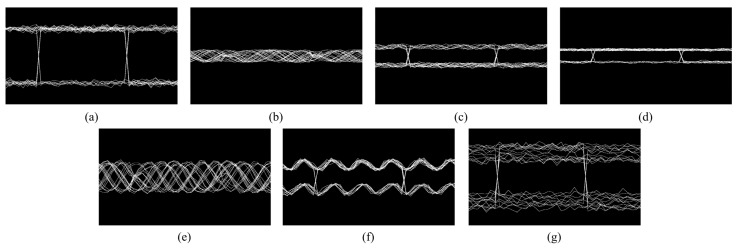
Eye diagrams under various types of interference: (**a**) clean signal, (**b**) CWI, (**c**) chirp interference, (**d**) pulse interference, (**e**) FMI, (**f**) AMI, and (**g**) spoofing interference.

An eye diagram is a two-dimensional representation of the time domain superposition of the output sequence of the GNSS receiver baseband correlator. Its shape is essentially a statistical visualization of signal integrity and uncertainty (entropy) within the pseudocode cycle. Interference signals destroy the correlation peak structure (amplitude, phase, and timing dimensions) and produce characteristic distortion patterns in the eye diagram shape (Figure 2).

## 3. ICOA–CNN–ResNet–BiLSTM Interference Classification Method

**Figure 3:**
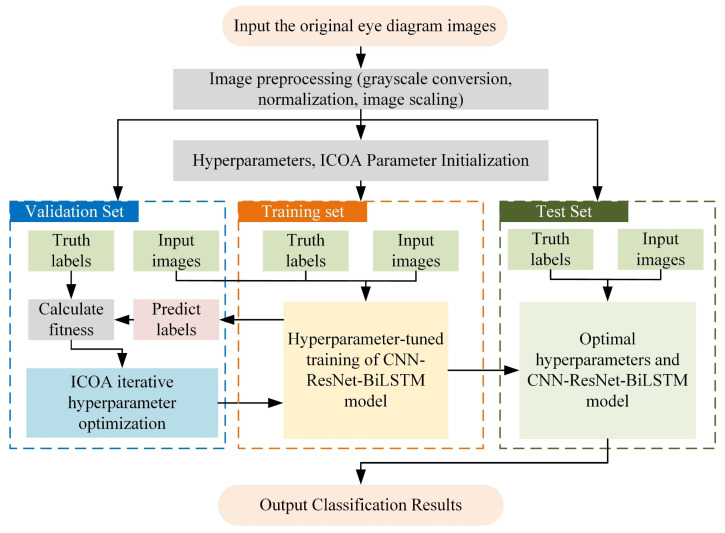
CNN–ResNet–BiLSTM interference signal classification model optimized on the basis of the ICOA.

In this study, the classification problem for suppression and deception interference signals is addressed by proposing an ICOA–CNN–ResNet–BiLSTM hybrid model (Figure 3). First, the original images are preprocessed by grayscaling, normalization, and scaling to extract the key features of the interference signals. Then, the processed data are divided into training, validation, and test sets for model training, hyperparameter tuning, and performance evaluation, respectively. In the model training stage, a hybrid architecture that combines a CNN, a ResNet, and BiLSTM is constructed to leverage their respective advantages in spatial feature extraction and temporal modeling. To improve model performance, key hyperparameters (such as the learning rate, number of neurons, and number of convolutional kernels) are iteratively optimized via the ICOA. The ICOA evaluates the difference between the prediction results and the actual labels on the validation set, calculates the fitness function, and dynamically adjusts the hyperparameter configuration to effectively avoid falling into local optima and enhance the model’s generalizability. Finally, the optimized hyperparameters are applied to the hybrid model to explore the deep spatial and temporal features of the interference signals fully. After the model training is complete, the test set is used to verify the performance, and the classification effect is evaluated in terms of metrics such as accuracy, recall, and the F1 score. The experimental results demonstrate that this method not only improves hyperparameter search efficiency but also effectively integrates spatial, temporal, and global features, thereby significantly enhancing the accuracy and robustness of deception interference signal recognition.

### 3.1. Image Preprocessing

To prepare the raw input images (initial dimensions: 1500×844×3) for efficient and effective analysis by the subsequent deep learning model, a focused three-stage preprocessing pipeline is applied. This pipeline is designed to: (1) grayscale conversion: remove color channel dependencies and reduce noise/complexity, (2) normalization: standardize the input intensity range to enhance model stability and sensitivity to subtle features, and (3) image resizing: reduce the spatial resolution significantly to improve computational efficiency for model training and inference while preserving critical spatial patterns. Overall, this preprocessing pipeline ensures that the model receives clean, standardized, and computationally manageable inputs optimized for learning and inference.

- Grayscale Conversion

RGB images are transformed into single-channel grayscale representations via the luminosity method, which is defined as follows:

(6) $$I_{g}=0.299R+0.587G+0.114B$$

where R, G, and B denote the red, green, and blue channel intensities, respectively. This operation reduces the data dimensionality from 1500×844×3 to 1500×844×1 and eliminates color interference while preserving the spatial relationships that are critical for signal characterization.

- Normalization

Pixel intensity values are scaled to the range [0, 1] via min–max normalization:

(7) $$I_{n}=\frac{I_{g}-I_{\min}}{I_{\max}-I_{\min}}$$

where $I_{g}$ and $I_{\min}$ represent global extrema across the 1500×844×1 grayscale image. This step enhances contrast sensitivity for low-amplitude interference components.

- Image Resizing

Grayscale images are resampled to a standardized resolution of 128×128×1 via bilinear interpolation:

(8) $$I_{r}=\mathrm{resize}\left(I_{n},(128,128)\right)$$

This spatial reduction (horizontal: ≈11.7× and vertical: ≈6.6×) optimizes computational efficiency while preserving the structural continuity of interference patterns through adaptive bilinear weighting.

The input is downsampled from 1500×844×3 RGB to 128×128×1 grayscale, thereby achieving 98.7% compression and an improved signal-to-noise ratio for more effective feature extraction.

### 3.2. CNN–ResNet–BiLSTM

**Figure 4:**
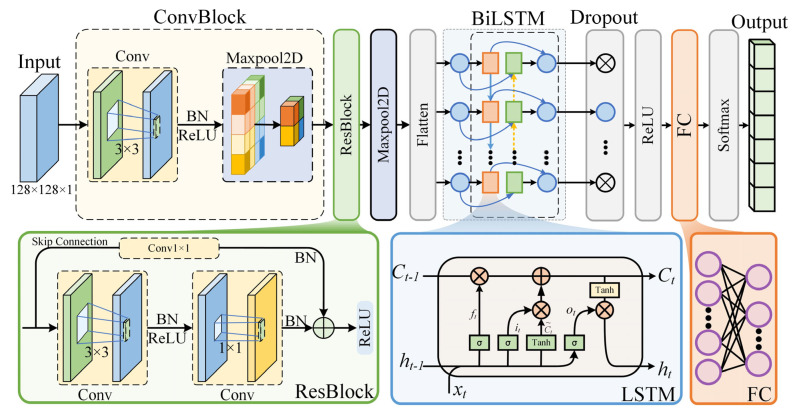
CNN–ResNet–BiLSTM model structure.

The CNN–ResNet–BiLSTM hybrid model fully integrates the advantages of the three network structures in spatial feature extraction and temporal modeling to achieve synergistic enhancement. The model (Figure 4) consists of the following core modules and functions.

- CNN feature extraction layer

The model first applies a two-dimensional convolution (2D-CNN) operation to the input GNSS eye diagram image to extract interference features with local spatial characteristics in the image. Convolution operations can effectively identify short-term interference patterns (such as short-term bursts and periodic textures) and can be combined with pooling operations (such as MaxPooling) to further compress the feature dimensions and enhance robustness.

- Residual network module (ResBlock)

To deepen feature representation, a residual module is introduced, which includes two convolution layers and a skip connection layer. By setting multiscale convolution kernels, the model can accommodate interference features at various scales, which improves its ability to recognize complex interference types. Moreover, the residual structure effectively alleviates the gradient disappearance and performance degradation problems in deep networks, thereby ensuring the stable transmission of features in deep networks.

- BiLSTM sequence modeling layer

After being processed by the residual module, the high-order features are first flattened into a sequence format via a max pooling layer and a flatten layer and then input into the BiLSTM layer. BiLSTM possesses forward and backward temporal dependency modeling capabilities, which enable it to capture dynamic changes in signals across the temporal dimension. This approach is particularly effective for identifying interference types with temporal evolution characteristics, thereby significantly enhancing the model’s recognition accuracy and robustness against dynamic interference.

- Classification layer

The output of the BiLSTM, which consists of a fully connected layer (FC) and a SoftMax layer, is mapped to a predefined interference category space via the FC layer, and the SoftMax layer outputs the predicted probability of each interference category, thus completing the final classification task.

- Normalization and regularization

To accelerate the training process, improve model stability, and prevent overfitting, multiple batch normalization (BN) layers and dropout layers are employed in the model. During training, the Adam optimizer is used in combination with a learning rate decay mechanism to further improve training stability and model generalizability. Additionally, ReLU activation functions are employed in all the network layers to enhance the model’s nonlinear expression capability and accelerate convergence.

### 3.3. ICOA

**Figure 5:**
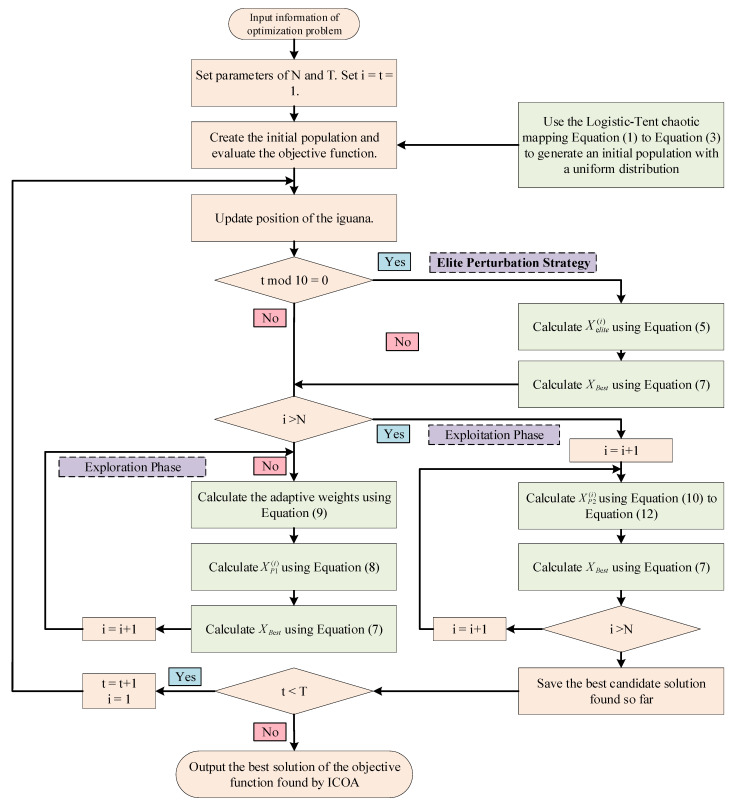
ICOA Process.

To address the inherent shortcomings of the COA in high-dimensional complex search spaces, such as becoming stuck in local optima and insufficient convergence accuracy, the proposed ICOA optimizes the existing COA through three core improvements: (1) In the population initialization stage, the traditional random initialization strategy is replaced with a logistic–tent chaotic mapping that combines the characteristics of logistic mapping and tent mapping, which significantly improves the uniformity and exploration of the initial population and lays a foundation for diversity in global search; (2) an elite perturbation strategy (EPS) is introduced, which applies periodic, adaptively decaying perturbations to the current optimal solution (elite individuals) and uses a greedy selection mechanism to dynamically inject controllable randomness to effectively escape from local optima; and (3) the dynamically decaying weight coefficients are dynamically optimized between the exploration and exploitation phases, which includes adjusting exploration intensity during the exploration phase and improving the original linear boundary contraction strategy to a nonlinear power-law contraction strategy with elite guidance during the exploitation phase, thereby collectively enhancing the algorithm’s global exploration capability and local optimization efficiency in complex search spaces. The process of the ICOA (Figure 5) is as follows.

#### 3.3.1. Logistic–Tent Chaotic Mapping

To address the issues of uneven population distribution and insufficient coverage of the solution space that may arise with traditional random initialization methods in the COA, an improved strategy—logistic–tent chaotic mapping initialization—is employed in this study. By combining the traversal characteristics of logistic mapping and tent mapping, this method significantly enhances the uniformity and diversity of the initial population, thereby effectively avoiding local convergence and population clustering during the search process. The mathematical expression for the logistic map is as follows:

(9) $$x_{Logistic}^{\left(k+1\right)}=3.9x_{Logistic}^{\left(k\right)}\cdot \left(1-x_{Logistic}^{\left(k\right)}\right)$$

where $x_{Logistic}^{0}\sim U\left(0,\;1\right)$, k represents the number of mappings, and $x_{Logistic}^{\left(k+1\right)}$ represents the function value of the k-th mapping.

The mathematical expression for the tent map is as follows:

(10) $$x_{Tent}^{\left(k+1\right)}=\left\{\begin{array}{c} 1.999x_{Tent}^{\left(k\right)},\;\;\;\;\;\;x_{Tent}^{\left(k\right)}<0.5\\ 1.999\left(1-x_{Tent}^{\left(k\right)}\right),\;\;\;\;\;otherwise \end{array}\right.$$

where $x_{Tent}^{0}\sim U\left(0,\;1\right)$, k represents the number of mappings, and $x_{Tent}^{\left(k+1\right)}$ represents the function value of the k-th mapping.

The mathematical expression for the logistic–tent chaotic map is as follows:

(11) $$X^{\left(k\right)}=\mathrm{mod}\left(x_{Logistic}^{\left(k\right)}+x_{Tent}^{\left(k\right)},\;1\right)\left(ub-lb\right)+lb$$

where $X^{\left(k\right)}$ represents the population individual obtained by mapping back to the individual search space and where $\left[lb,ub\right]$ represent the upper and lower bounds of the solution space.

#### 3.3.2. Elite Perturbation Strategy

In the improved coati optimization algorithm, we adopted the EPS to enhance the algorithm’s ability to escape from local optima. The core idea of this strategy is to periodically apply controllable perturbations to the current optimal solution during the algorithm iteration process. By introducing moderate randomness, the algorithm breaks out of the local stable state into which it may fall while maintaining the trend of converging to the global optimal region. Elite perturbations are triggered periodically:

(12) $$Trigger\;Condition\left\{\begin{array}{c} Trigger,\;\;\;\;t\;\mathrm{mod}\;10\;=\;0\;\\ Do\;not\;Trigger,\;\;\;others \end{array}\right.\;$$

where t represents the current iteration number. To balance exploration and exploitation in the optimization process, we propose an elite adaptive perturbation strategy, where the intensity of the adaptive perturbation decays dynamically with the iteration process. In the early stages, local optima are escaped from by means of large perturbations. In the later stages, the perturbations are reduced to small perturbations to achieve accurate optimization. The adaptive perturbation expression is as follows:

(13) $$X_{elite}=X_{best}+\alpha \cdot \left(1-\frac{t}{T}\right)\cdot (ub-lb)\cdot (rand-0.5)$$

where $X_{best}$ represents the current globally optimal solution, α represents the basic disturbance coefficient (set to 0.1), T represents the maximum number of iterations, and rand represents a random vector whose entries are uniformly distributed in the interval [0, 1]. We impose boundary constraints on the disturbance:

(14) $$X_{elite}=\max(\min(X_{elite},ub),lb)$$

In addition, we also apply elite perturbation and greedy selection strategies for exploration and exploitation, which is expressed as

(15) $$X_{best}=\left\{\begin{array}{ll} X_{elite/P1/P2}, & \;F_{elite/P1/P2}<F_{best}\\ X_{best},\;\;\; & \;otherwise \end{array}\right.$$

After each solution is generated, if the current solution is better than the current optimal solution, the current optimal solution is replaced; otherwise, the original optimal solution is retained. The elite perturbation strategy injects vitality into the algorithm to escape from local optima while maintaining the convergence direction of the algorithm.

#### 3.3.3. Exploration and Exploitation Optimization

We introduce a dynamic weighting mechanism that is based on the original exploration and development stage to balance global exploration and local development capabilities. The weight decreases nonlinearly with the number of iterations, which enhances exploration capabilities in the early stages and focuses on development accuracy in the later stages.

(16) $$X_{P1}^{\left(i\right)}=\omega (t)\cdot X^{\left(i\right)}+\eta \cdot \left(X_{best}-I\cdot X^{\left(i\right)}\right)$$

where η~U(0,1) represents a uniformly distributed random number, I∈1,2 represents a randomly switched exploration direction coefficient, and ω is the following adaptive weighting function:

(17) $$\omega (t)=\omega_{max}-(\omega_{max}-\omega_{min})\cdot {\left(\frac{t}{T}\right)}^{2}$$

where $\omega_{max}=0.9$ and $\omega_{min}=0.2$.

During the development phase, we improved the original linear boundary contraction to a nonlinear power-law contraction and introduced an elite guidance mechanism to accelerate local convergence while preventing premature convergence. The nonlinear power-law contraction expression is as follows:

(18) $$\left\{\begin{array}{c} LO_{LOCAL}=lb\cdot \left(1-{\left(\frac{t}{T}\right)}^{k}\right)\\ HI_{LOCAL}=ub\cdot \left(1-{\left(\frac{t}{T}\right)}^{k}\right) \end{array}\right.$$

where k=2 is the nonlinear contraction index. The elite guidance mechanism can be expressed as

(19) $$X_{P2}^{\left(i\right)}=\left\{\begin{array}{ll} X^{\left(i\right)}+\Delta \;, & \;\;\rho >0.5\\ X_{best}+\Delta \;, & \;\mathrm{otherwise} \end{array}\right.$$

(20) $$\Delta =(1-2\zeta )\cdot \left(LO_{LOCAL}+\xi \cdot (HI_{LOCAL}-LO_{LOCAL})\right)$$

where ζ,ξ~U(0,1) represents an independent random number and where ρ~U(0,1) represents the probability of elite guidance selection.

#### 3.3.4. Performance Analysis

Four classic optimization test functions (the Rastrigin, Rosenbrock, Ackley, and Schwefel functions) were used as benchmarks (Table 2) to comprehensively evaluate the performance of the LCAVOA. These functions each have their own distinctive characteristics (such as strong oscillations, coexistence of flat regions and steep valleys, and narrow flat valleys), which were used to systematically examine the algorithm’s global search capability, convergence behavior, and solution accuracy. By comparing it with the APSO algorithm, CSA, SA algorithm, GWO algorithm, WOA, and COA, we analyzed the performance of the ICOA in terms of accuracy, speed, and stability. All the experiments were conducted under uniform conditions: a population size of 60, a maximum of 200 iterations, and 50 dimensions.

**Figure 6:**
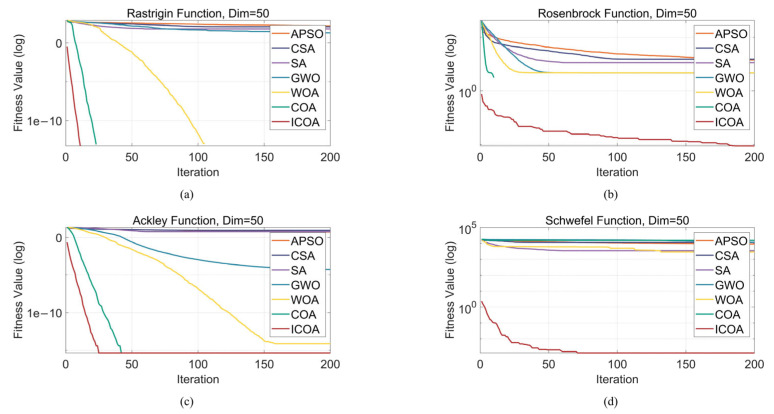
Fitness curves of seven algorithms on the Rastrigin, Rosenbrock, Ackley, and Schwefel functions. (**a**) Rastrigin function; (**b**) Rosenbrock function; (**c**) Ackley function; (**d**) Schwefel function.

**Figure 7:**
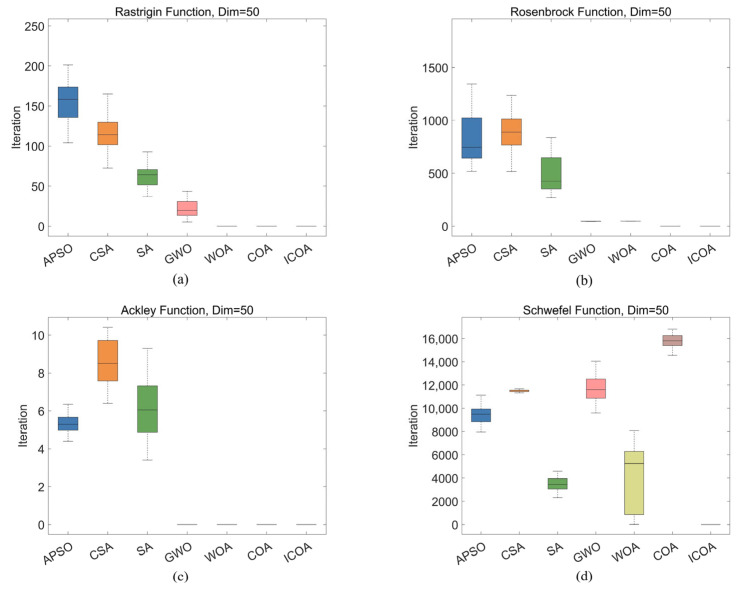
Optimal value distributions of seven algorithms on the Rastrigin, Rosenbrock, Ackley, and Schwefel functions. (**a**) Rastrigin function; (**b**) Rosenbrock function; (**c**) Ackley function; (**d**) Schwefel function.

The fitness curves of the optimization algorithms on the Rastrigin, Rosenbrock, Ackley, and Schwefel functions effectively demonstrate their performance differences, particularly in terms of global search capability, ability to escape from local optima, and convergence speed (Figure 6). To further compare the performance of these algorithms in a more intuitive manner, we visualized the solutions of each algorithm via box plots (Figure 7), which helped reveal the optimization effectiveness of the algorithms under various objective functions.

In the Rastrigin function optimization problem, the global search capability of the algorithm is crucial. Specifically, the APSO algorithm, CSA, SA algorithm, and GWO algorithm may easily became stuck in local optima during the optimization process, thereby resulting in suboptimal search results, with the optimal solution falling between 10^1^ and 10^2^. In contrast, the optimal solutions found by the WOA, COA, and ICOA were in the range of 10^−13^. However, the WOA had slower convergence, especially in the early stages, with a relatively slow decline in the fitness curve, thus failing to effectively accelerate convergence. In contrast, the COA demonstrated strong global search capabilities, which enabled it to converge quickly to optimal solutions, thus making it highly effective in solving the Rastrigin function optimization problem. Furthermore, by improving the search strategy, the ICOA outperformed the COA in terms of convergence speed and stability, converging in just 10 iterations. This indicated that its optimization performance was enhanced in multiple aspects, with a better balance between global exploration and local exploitation during the search process, thereby improving solution accuracy and algorithm convergence speed, achieving a 44% improvement in convergence speed compared to the original algorithm.

In the optimization of the Rosenbrock function, there is only one globally optimal solution, but its elongated shape requires the algorithm to perform precise searches along fine paths. APSO and the CSA performed the worst, with optimal solutions above 500, which deviated significantly from the true optimal solution; SA also performed poorly, with optimal solutions that fluctuated around 500; GWO and the WOA performed moderately, with the optimal solution at the 10^2^ level; the COA became stuck in a local optimum, with the optimal solution remaining at the 10^2^ level; and the ICOA demonstrated the best performance, with the optimal solution approaching the 10^−9^ level.

In the optimization of the Ackley function, the function features flat regions and steep valleys, with multiple local optima, which necessitates an algorithm with strong global search capabilities. The ICOA performed the best, with the optimal solution approaching the 10^−16^ order of magnitude, which was the closest to the ideal solution of 0; the COA’s optimal solution was of the same order of magnitude as that of the ICOA, but the COA required 17 more iterations to converge, with ICOA achieving a 40% improvement in convergence speed; the WOA’s optimal solution was on the 10^−15^ order of magnitude, but the WOA converged more slowly, requiring more than 150 iterations to converge; and the CSA, APSO, and SA performed similarly, with optimal solutions that ranged between 5 and 10.

Owing to the complex multipeaked nature of the Schwefel function, optimization algorithms face greater challenges. The optimal solutions of the CSA, GWO algorithm, and COA all exceeded 10,000 and were far from the ideal solution of 0; the optimal solutions of the APSO algorithm, SA algorithm, and WOA remained in the 10^3^–10^4^ range; and the ICOA achieved an optimal solution in the 10^−4^ range, thus performing the best.

In summary, the algorithms exhibited significant differences in terms of global search capability and convergence performance across various optimization problems. For the Rastrigin function optimization problem, the algorithms tended to become stuck in local optima during global search, which led to suboptimal optimization results. However, the COA and ICOA, with their strong global search capabilities, quickly converged to optimal solutions. In particular, the ICOA, through improved search strategies, demonstrated superior performance in terms of convergence speed and stability, with better optimization performance than the COA, achieving a more than 40% improvement in convergence speed. For the Rosenbrock function, although APSO and the CSA performed poorly, with their optimal solutions far from the true optimal solution, the ICOA demonstrated advantages in fine-grained search and obtained a solution that was close to the true optimal solution. In the optimization of the Ackley function, the global search capability of the ICOA also played an important role, and it obtained the results closest to the ideal solution. Other algorithms, such as the COA and WOA, although obtaining results similar in magnitude to those obtained by the ICOA, converged more slowly. Owing to its complex multipeaked nature, the Schwefel function is difficult to optimize, but ICOA still performed the best, as it was the only one to obtain an optimal solution in the 10^−4^ order of magnitude, while other algorithms had optimal solutions in the 10^4^ order of magnitude. In conclusion, the ICOA demonstrates strong global search capabilities and excellent convergence performance across various test functions,. It effectively converges in several test functions, exhibiting strong stability, thus making it a reliable optimization method.

## 4. Interference Signal Classification Performance Test

### 4.1. Interference Signal Dataset

In this study, a mixed dataset that consisted of two sources was used for interference classification tasks. The spoofing interference signals were from the TEXBAT [32] dataset developed by the University of Texas at Austin, which was designed specifically for GNSS spoofing research and covers 10 types of typical spoofing scenarios, including dynamic/static targets, matched-power/suppression, carrier and code phase locking/nonlocking, Doppler frequency matching, and signal structure replication attacks (such as code estimation and replay timing attacks). Suppression interference signals were generated via GNSS signal simulation software (SoftGNSS v3.0) in five major interference categories, namely, single-tone/multitone CWI, linear/quadratic/logarithmic sweep chirp interference, pulse interference, FMI, and AMI, with a jamming-to-signal ratio that ranged from 10 dB to 30 dB.

On the basis of the above signal sources, 7048 eye diagrams were constructed through the receiver correlator output to characterize different interference patterns, and the interference labels were labeled (Table 3). A strict random splitting strategy was employed to divide the dataset into a training set (5638 frames), a validation set (705 frames), and a test set (705 frames) at an 8:1:1 ratio to ensure the effectiveness of model training and the generalization ability of the evaluation.

### 4.2. Classification Evaluation Metrics and Performance Assessment

#### 4.2.1. Evaluation Metrics: Definitions and Calculation Methods

To analyze the performance characteristics of the classification model in depth, we constructed a multidimensional evaluation framework that covered the following key metrics:

- Accuracy

Accuracy represents the proportion of correct predictions made by the model and measures its basic discriminability. The calculation formula is

(21) $$Accuracy=\frac{TP+TN}{TP+TN+FP+FN}$$

where the number of true positives (TP) represents the number of correctly predicted positive examples, the number of true negatives (TN) represents the number of correctly predicted negative examples, the number of false positives (FP) represents the number of negative examples incorrectly predicted as positive, and the number of false negatives (FN) represents the number of positive examples incorrectly predicted as negative.

- Precision

Precision is used to assess the reliability of a model’s prediction when it predicts that a sample is positive. The calculation formula is

(22) $$Precision=\frac{TP}{TP+FP}$$

- Recall

Recall describes extent to which a model covers or captures positive samples that actually exist. The calculation formula is

(23) $$Recall=\frac{TP}{TP+FN}$$

- Specificity

Specificity reflects the ability of a quantitative model to correctly exclude negative samples that actually exist. The calculation formula is

(24) $$Specificity=\frac{TN}{TN+FP}$$

- F1 Score

Precision and recall are integrated into a single metric, namely, the F1 score, for evaluating the overall performance of a model in positive class identification, especially when there is a trade-off between precision and recall. The calculation formula is

(25) $$F1\;Score=\frac{2\cdot \left(Precision\cdot Recall\right)}{Precision+Recall}$$

#### 4.2.2. Interference Signal Classification Performance Verification

The experiments were conducted on a Windows 11 platform equipped with an Intel Core i7-9700k processor (3.6 GHz, manufactured by Intel, USA), 32 GB of DDR4 memory (manufactured by Samsung, Korea), and an NVIDIA RTX 3070 Ti graphics card (manufactured by NVIDIA, USA). The comparison model framework consisted of four levels: (1) traditional machine learning (SVM and MLP); (2) basic deep learning units (LSTM, BiLSTM, CNN, and GRU [33]); (3) composite structures (CNN-BiLSTM [34] and CNN-LSTM); and (4) the deep hybrid model ICOA–CNN–ResNet–BiLSTM.

Each model was trained for 60 rounds, with the number of hidden units set to 128. For the ICOA–CNN–ResNet–BiLSTM model, the evolutionary population size was set to 2, and the iteration count was set to 2. The hyperparameters of the ICOA–CNN–ResNet–BiLSTM model were determined through systematic optimization using the ICOA. The optimized hyperparameter configuration is as follows: a learning rate of 0.0009; a channel configuration of the convolutional module set to (64–64–64); a dual convolutional structure with channel counts of 64 and 64 and 64 skip connections adopted for the residual main path; and the number of hidden units in the BiLSTM set to 128.

**Figure 8:**
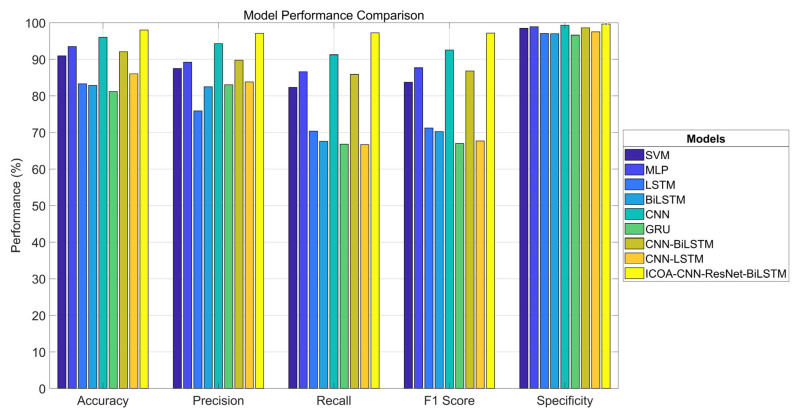
Comparison of interference signal classification performance among various models (visualization).

Table 4 and Figure 8 present the experimental results of various classification models on the TEXBAT test set. The models exhibited significant differences in terms of accuracy, precision, recall, F1 score, and specificity, which indicate disparities in their classification capabilities and generalization performance for interference signal identification.

Between the traditional machine learning methods, the SVM performed the best, with an accuracy of 90.95% and a specificity of 98.44%. However, its recall rate was only 82.30%, which indicates that there were false negatives. In contrast, the MLP improved on all the metrics, with an accuracy of 93.49%, a recall rate of 86.59%, an F1 score of 87.66%, and a specificity of 98.89%, thus exhibiting a more balanced performance and stronger generalization ability.

Among the basic deep learning models, the CNN led with an accuracy rate of 96.04% and an F1 score of 92.55%, thus verifying its advantages in local feature extraction in images. In comparison, the time series models (LSTM, BiLSTM, and GRU) performed poorly overall, with accuracy rates below 85%, and the GRU had the lowest accuracy of only 81.18%. Although these models are theoretically applicable to time series data, their training effectiveness is limited on image data. In terms of F1 score, LSTM and BiLSTM achieved 71.22% and 70.23%, respectively. While the GRU had a higher accuracy rate (83.04%), its recall rate was only 66.80%, which resulted in an F1 score of 67.00%.

Among the hybrid models, CNN-BiLSTM performed the best, with an accuracy rate of 92.07%, an F1 score of 86.80%, and a recall rate of 85.88%. Although it was slightly outperformed by the CNN alone, it was still competitive overall. CNN-LSTM performed poorly, with a recall rate of only 66.67% and an F1 score of 67.68%, which were close to those of the GRU, thus indicating that there is still room for optimization in the fusion design.

The optimized model proposed in this study, namely, ICOA–CNN–ResNet–BiLSTM. significantly outperformed the other methods in terms of all the other metrics; it achieved accuracy, precision, recall, F1 score, and specificity scores of 98.02%, 97.09%, 97.24%, 97.14%, and 99.65%, respectively, which were 1.98%, 2.80%, 6.10%, 4.59%, and 0.33% higher than those of the second-best model. This model integrates the structural advantages of feature optimization (ICOA), CNN, ResNet, and BiLSTM to balance accuracy and stability and significantly enhance recognition capabilities while reducing the risk of missed detections.

In summary, the deep fusion model (ICOA–CNN–ResNet–BiLSTM) performed best in interference signal classification and significantly outperformed traditional methods and single deep models in processing entropy-rich interference signals. The CNN single-model structure demonstrated robust performance, whereas temporal (LSTM-based) models have limited applicability to image data.

**Figure 9:**
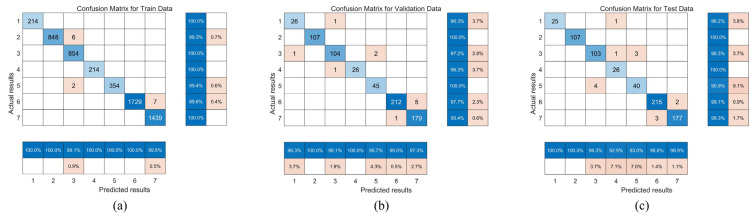
Confusion matrices for ICOA–CNN–ResNet–BiLSTM classification: (**a**) Training set, (**b**) validation set, and (**c**) test set.

We plotted the confusion matrices of the classification results of the ICOA–CNN–ResNet–BiLSTM model on three datasets (Figure 9). Each cell in the confusion matrix represents a match between the actual category and the predicted category, where the numbers on the diagonal represent the numbers of correctly classified samples and the numbers off the diagonal represent the numbers of misclassified samples. In addition, the corresponding accuracy and error rate were calculated for each category to more intuitively evaluate the model’s performance on each category.

Taking the confusion matrix of the test set in Figure 9c as an example, there are 107 samples in category 3 (CWI), of which 103 were correctly classified by the model, and only 4 samples were incorrectly identified as category 5 (pulse interference), which indicates that there may be feature overlap or low distinguishability between pulse interference and CWI. Further analysis of the classification performance of other categories reveals that the model performed exceptionally well in most categories, particularly in categories 1 (AMI), 2 (Chirp interference), 6 (spoofing interference), and 7 (clean and interference-free signal), where the accuracy rates exceeded 98.5%, which demonstrates the model’s strong discriminative ability in handling such signals.

However, the model’s classification accuracy was relatively low for categories 4 (FMI) and 5 (pulse interference), which indicates that there are still challenges in identifying these categories. This may be attributable to high interclass similarity among samples, overlapping feature distributions, or insufficient training samples. Therefore, future research could focus on enhancing data diversity and optimizing the feature extraction network to further improve the model’s ability to identify easily confused categories.

## 5. Conclusions

In this study, an ICOA-based CNN–ResNet–BiLSTM hybrid deep learning architecture for high-precision classification of GNSS interference signals is proposed. In this architecture, a complete processing flow that includes eye diagram feature space transformation, deep model training, and global hyperparameter optimization (implemented by ICOA) is constructed. This integrated approach enables the model to effectively capture and analyze the high information entropy inherent in complex interference environments. In the interference signal classification task, the proposed hybrid model has significant performance advantages. The conclusions drawn from this study are as follows:

1. The proposed ICOA demonstrates outstanding performance in model hyperparameter optimization. In a systematic evaluation of four classic test functions (Rastrigin, Rosenbrock, Ackley, and Schwefel), the ICOA demonstrated significant advantages over mainstream optimization algorithms (such as APSO, the CSA, SA, GWO, the WOA, and the COA) in terms of global search capability, convergence speed, and accuracy. For example, on the Rastrigin function, the algorithm successfully avoided becoming trapped in local optima and achieved optimization results at the 10^−13^ magnitude level, with its convergence speed improved by more than 30% compared with that of the next-best algorithm. On the Rosenbrock function, it achieved high-precision convergence (reaching the 10^−9^ order of magnitude) and outperformed the next-best algorithm by nine orders of magnitude. On the multipeak Ackley function, it achieved 100% global convergence with 17 fewer iterations than the COA. On the high-dimensional complex Schwefel function, it outperformed the next-best algorithm by 10^7^ times in terms of the optimization results. The above performance improvements were due mainly to the integration of three strategies in the ICOA, namely, logistic–tent chaotic mapping, an elite perturbation mechanism, and dynamic weight adjustment, which significantly enhance the balance between population diversity and exploration–exploitation. The results show that ICOA, which is a highly robust optimizer, can provide stable and efficient support for the parameter configuration of complex deep models and has good generalization potential and engineering adaptability.
2. In this study, the performance of multiple models in interference signal classification tasks was verified. The results show that the deep fusion model ICOA–CNN–ResNet–BiLSTM significantly outperformed traditional methods and single deep learning structures in terms of all the metrics. The model achieved the highest scores in terms of accuracy, precision, recall, F1 score, and specificity, with values of 98.02%, 97.09%, 97.24%, 97.14%, and 99.65%, respectively, which were improvements of 1.98%, 2.80%, 6.10%, 4.59%, and 0.33%, respectively, over those of the next-best model, thus demonstrating excellent classification ability and robustness. This comparison shows that although traditional models such as SVM and MLP have certain advantages in terms of specific metrics and CNN perform well in basic deep learning models, time series models (such as LSTM networks and GRUs) have limited effectiveness on image data. Although hybrid structures have potential, they still rely on optimized designs. Overall, models that integrate feature optimization mechanisms with multilayer deep structures demonstrate significant advantages in improving recognition accuracy and reducing false negative rates, thus providing more reliable solutions for interference signal recognition.
3. An eye diagram-enabled intelligent interference recognition paradigm was innovatively constructed. The high-confidence classification results generated by this framework can provide core decision support for dynamic anti-interference strategies (such as adaptive notch filtering and space–time filtering) for receivers, increase the resilience of GNSSs in complex electromagnetic environments, and provide lightweight solutions for infrastructure security protection.

## Figures and Tables

**Table 1:** Types of interference signals and their mathematical models.

Interference Type		Formula	Explanation
CWI	single tone (N = 1)	$j_{CW}(t)=\sum_{j=1}^{N}A_{j}\cos(2\pi f_{c}t+\phi_{0})$	Interference caused by the superposition of one or more sine waves, where $A_{j}$ represents the signal amplitude, $f_{c}$ represents the center frequency, $\phi_{0}$ represents the initial phase, and t represents the time variable.
	multitone (N > 1)		
Chirp interference	linear sweep	$\begin{array}{l} J_{Sweep}\left(t\right)=A_{j}\cos\left(2\pi \left(f_{0}+\frac{\left(f_{1}-f_{0}\right)}{t_{1}}t\right)t+\phi_{0}\right) \end{array}$	The frequency of the second sweep signal varies with time according to a quadratic curve (parabola), where $f_{0}$ represents the initial frequency, $f_{1}$ represents the final frequency, t represents the time variable (range [0, $t_{1}$]), and $t_{1}$ represents the time point at which the sweep ends.
	quadratic sweep	$\begin{array}{l} J_{Sweep}\left(t\right)=A_{j}\cos\left(2\pi \left(f_{0}+\frac{\left(f_{1}-f_{0}\right)}{t_{1}^{2}}t^{2}\right)t+\phi_{0}\right) \end{array}$	The frequency varies with time according to a quadratic function.
	logarithmic sweep	$\begin{array}{l} J_{Sweep}\left(t\right)=A_{j}\cos\left(2\pi \left(f_{0}{\left(\frac{f_{1}}{f_{0}}\right)}^{\frac{t}{t_{1}}}\right)t+\phi_{0}\right) \end{array}$	The frequency of a logarithmic sweep signal varies logarithmically with time.
Pulse interference		$\begin{array}{l} J_{Pulse}\left(t\right)=A_{j}\sum_{k}p\left(t-kT_{p}\right)\cos\left(2\pi f_{j}t\right) \end{array}$	Periodic pulse signal, where $A_{j}$ represents the signal amplitude, p(t) represents the baseband pulse, $T_{p}$ represents the period, and modulation is performed by using the carrier frequency $f_{j}$.
FMI		$\begin{array}{l} J_{FM}\left(t\right)=A_{j}\cos\left(2\pi f_{j}t+\beta \int_{0}^{t}n\left(\tau \right)d\tau \right) \end{array}$	Modulates the carrier frequency $f_{j}$ with the noise signal $n\left(\tau \right)$, where $A_{j}$ represents the signal amplitude and β represents the modulation index.
AMI		$\begin{array}{l} J_{AM}\left(t\right)=\left[1+m\cdot n\left(t\right)\right]\cdot A_{j}\cos\left(2\pi f_{c}t\right) \end{array}$	Modulates the carrier amplitude $f_{j}$ with noise signal $n\left(\tau \right)$, where $A_{j}$ represents the modulation depth and β represents the center frequency.
Spoofing interference		$J_{Spoof,i}(t)=A_{j,i}\cdot d_{Spoof,i}(t)\cdot c_{i}\left(t-\tau_{i}(t)\right)\cdot \cos\left(2\pi (f_{i}+\Delta f_{i})t+\phi_{i}\right)$	Where $A_{j,i}$ represents the amplitude of the i-th fake satellite signal, $d_{Spoof,i}(t)$ represents a fake navigation message, $c_{i}\left(t\right)$ represents pseudorandom code (C/A code), $\tau_{s}$ represents the satellite-specific time delay, $\Delta f_{i}$ represents the Doppler frequency shift, and $\phi_{i}$ represents the initial phase.

**Table 2:** Theoretical properties of the test functions.

Function	Ideal Optimal Value	Search Range	Optimal Solution Location	Core Challenges
Rastrigin: $f\left(x\right)=\sum_{i=1}^{n}\left(x_{i}^{2}-10\cdot \cos\left(2\pi x_{i}\right)\right)+10n$	0	[−5.12, 5.12]	Center	Strong oscillations, dense local optima, tests escape from premature convergence
Rosenbrock: $f\left(x\right)=\sum_{i=1}^{n-1}\left[100\cdot {\left(x_{i+1}-x_{i}^{2}\right)}^{2}+{\left(1-x_{i}\right)}^{2}\right]$	0	[−5, 10]	Center	Nonconvex, narrow flat valleys, gradient directions mislead the search process
Ackley: $\begin{array}{l} f\left(x\right)=-20\cdot \exp\left(-0.2\cdot \sqrt{\frac{1}{n}\sum_{i=1}^{n}x_{i}^{2}}\right)\\ \;\;\;-\exp\left(\frac{1}{n}\sum_{i=1}^{n}\cos\left(2\pi x_{i}\right)\right)\\ \;\;\;+20+\exp\left(1\right) \end{array}$	0	[−32.768, 32.768]	Center	Flat regions and steep valleys coexist, balancing exploration and exploitation
Schwefel: $f\left(x\right)=418.9829n-\sum_{i=1}^{n}\left(x_{i}-420.9687\right)\sin\left(\sqrt{\left|x_{i}-420.9687\right|}\right)$	0	[−500, 500]	Boundary	Deceptively dense local optima (near the center), global optimum at the boundary, tests global search and boundary handling capabilities

**Table 3:** Types and categories of interference signals in the dataset.

Interference Category	Label
AMI	1
Chirp interference	2
CWI	3
FMI	4
Pulse interference	5
Spoofing interference	6
Clean and interference-free signal	7

**Table 4:** Comparison of interference signal classification performance among various models.

Model	Accuracy	Precision	Recall	F1 Score	Specificity
SVM	90.95%	87.51%	82.30%	83.73%	98.44%
MLP	93.49%	89.22%	86.59%	87.66%	98.89%
LSTM	83.31%	75.89%	70.37%	71.22%	97.06%
BiLSTM	82.88%	82.48%	67.58	70.23	96.98%
CNN	96.04%	94.29%	91.23%	92.55%	99.32%
GRU	81.18%	83.04%	66.80%	67.00%	96.62%
CNN-BiLSTM	92.07%	89.75%	85.88%	86.80%	98.60%
CNN-LSTM	85.99%	83.82%	66.67%	67.68%	97.52%
ICOA–CNN– ResNet–BiLSTM	98.02%	97.09%	97.24%	97.14%	99.65%

## Data Availability

The TEXBAT datasets are provided by the radio navigation laboratory of the University of Texas at Austin and can be downloaded via the following hyperlink: https://radionavlab.ae.utexas.edu/texbat (accessed on 1 December 2024).
